# Personalised Approach to the Management of Older People with Type 2 Diabetes Mellitus—A Comprehensive Narrative Review

**DOI:** 10.3390/jpm16040213

**Published:** 2026-04-13

**Authors:** Alan Sinclair, Mohammed Al-Banna, Roxana Tutunariu, Ahmed H. Abdelhafiz

**Affiliations:** 1 King’s College, London WC2R 2LS, UK; alan.sinclair@kcl.ac.uk; 2 Foundation for Diabetes Research in Older People (fDROP), Droitwich Spa WR9 0QH, UK; 3 Department of Geriatric Medicine, Rotherham General Hospital, Moorgate Road, Rotherham S60 2UD, UK; mohammed.al-banna@nhs.net (M.A.-B.); roxana.tutunariu@nhs.net (R.T.)

**Keywords:** older people, diabetes mellitus, frailty, management, personalised

## Abstract

The global population is ageing due to increased life expectancy, and the prevalence of diabetes is proportionally increasing. With advancing age, diabetes in older people is a complex condition due to associated morbidities and geriatric syndromes. As a result, the management of diabetes in old age is challenging. Due to the wide heterogeneity of older people, diabetes management in this age group should be personalised. While strict targets are accepted in fit individuals, relaxed targets should be considered in patients with multiple morbidities and a high risk of hypoglycaemia. The development of frailty changes the metabolic profile of older people, and their insulin resistance and diabetes trajectory, which will have an impact on the choice of glucose-lowering agents and the goals of therapy. For example, intensive therapy, the use of SGLT-2 inhibitors and GLP-1RA, and tight targets should be continued in frail, sarcopenic, obese individuals due to their increased insulin resistance and cardiovascular risk. On the other hand, relaxed targets and deintensification of therapy should be considered in anorexic, malnourished, frail individuals with significant weight loss due to their low insulin resistance, low prevalence of cardiovascular risk factors, and high risk of hypoglycaemia. Annual reviews of older people with diabetes should include screening for frailty, depression and dementia for early diagnosis, and appropriate interventions. The introduction of continuous glucose monitoring is increasingly used in older people with diabetes and has the potential to reduce the incidence of hypoglycaemia. With the expectation of a continued increase in the prevalence of older people with diabetes, the use of mobile health may allow care delivery on a wider scale without the need for face-to-face appointments. In addition, there is a promising scope for artificial intelligence to achieve better diabetes outcomes. Future research is still required to expand the use of these technologies in older age groups.

## 1. Introduction

Life expectancy is increasing, leading to an increased prevalence of older people living with diabetes mellitus. For instance, the current global prevalence of diabetes is 589 million adults (20–79 years old) and is expected to increase to 853 million by the year 2050, an increase of 46%. The current prevalence is highest among older people, with a peak of 24.79% in the age group 75–79 years old [1]. With an increasing duration of diabetes, old age is associated with an increasing prevalence of multiple morbidities due to the accumulation of diabetes-associated complications and diabetes-related morbidities, as well as age-related diseases such as osteoporosis, falls, injuries and dementia. In addition, frailty is increasingly recognised as an important and clinically significant complication of diabetes in older people [2]. This will have a significant impact on older people’s physical and mental function. Therefore, the phenotype of diabetes in old age is widely heterogeneous, from fit individuals independently living in the community to fully dependent housebound persons or residents in care homes with multiple disabilities. As a result, the management of diabetes in this age group is challenging given this heterogeneity and these complex needs. This manuscript comprehensively highlights the case mix of this population, reviews the clinical problems common in this age group, and explores a tailored management approach that may help to deliver personalised treatment based on individuals’ characteristics rather than chronological age.

## 2. Methods

This comprehensive narrative review involved searching three databases: Medline, Google Scholar, and PubMed, covering articles published from 1996 to the present. We employed Medical Subject Heading (MeSH) terms such as “elderly,” “older people,” “aged,” “frailty,” “frail,” “diabetes mellitus,” “management,” “glucose control,” “physical function,” “mental function,” “dementia,” “hypoglycaemia,” “continuous glucose monitoring,” “mobile health,” and “artificial intelligence.” We limited our selection to articles published in the English language. The authors initially screened articles for relevance based on abstracts. Furthermore, we conducted a manual search of citations within the retrieved articles to identify additional relevant studies beyond those captured in the electronic searches. In addition to database searches, we also examined diabetes and geriatric medicine organisations for articles on the management of frail older people with diabetes to ensure comprehensive coverage of the relevant literature. These organisations included the European Diabetes Working Party for Older People (EDWPOP), International Diabetes Federation (IDF), International Position Statement on the management of frailty in diabetes mellitus, and the European Union Geriatric Medicine Society (EuGMS). The authors are aware that this search strategy could be limited by missing contributions from non-English published articles. We resolved any disagreements by detailed discussion and review of any conflicts in data interpretation to reach mutual agreement.

## 3. Physical Dysfunction

The study of osteoporotic fractures research group showed that diabetes prospectively increased the risk of physical dysfunction twofold, after adjustment for confounding factors, compared to those without diabetes [3]. The yearly incidence of any functional disability was 9.8% among women with diabetes and 4.8% among women without diabetes [3]. The decline in physical function is likely due to multiple factors, including comorbidities, obesity, coronary heart disease, arthritis, diabetes-related complications such as peripheral neuropathy and visual impairment, the duration of diabetes, poor glycaemic control, nutritional deficiency, polypharmacy, and recurrent hypoglycaemia [3,4]. Physical dysfunction leads to impairment in performing activities of daily living (ADL), instrumental ADL (IADL), lower limb mobility, and general physical activities [5]. In a systematic review and meta-analysis of 26 (mostly cross-sectional) studies, diabetes was associated with an increased risk of mobility disability {odds ratio (OR) 1.71, 95% confidence interval (CI) 1.53 to 1.91}, IADL disability (1.65, 1.55 to 1.74), and ADL disability (1.82, 1.63 to 2.04) [6]. Functional independence is important for adherence to self-care tasks in older people with diabetes [7]. In addition, the high prevalence of physical disability places a substantial burden on patients themselves, their carers, and the health care system, as well as raising overall costs [8].

## 4. Mental Dysfunction

In older people, diabetes is associated with an elevated risk of mental function decline. Observational studies show that diabetes leads to an increased risk of Alzheimer’s disease (AD), relative risk (RR) 1.56 (95% CI 1.41 to 1.73), vascular dementia (2.27, 1.94 to 2.66), and all dementia types (1.73, 1.65 to 1.82). In addition, diabetes increases the risk of mild cognitive impairment (MCI) and its progression to dementia threefold [9,10,11]. The decline of cognitive function in diabetes is likely due to persistent hyperglycaemia, causing vascular complications, especially cerebrovascular disease, accelerated cerebral ageing and amyloid deposition, and repeated episodes of hypoglycaemia causing direct brain cell death [12,13,14]. The Health, Ageing and Body Composition Study showed that diabetes is prospectively associated with an increased risk of depression (hazard ratio (HR) 1.31, 95% CI 1.07 to 1.61) [15]. Anxiety, a condition that commonly co-exists with depression, is also common in diabetes (OR 1.99, 95% CI 1.22 to 3.25) [16]. The decline in mental function in diabetes is likely due to diabetes-related structural and functional brain changes in areas responsible for mood stabilisation, as well as hippocampal atrophy that leads to changes in mood [17,18]. Another important factor is the burden of diabetes diagnosis and disease-related complications and self-care tasks that lead to a depressed mood [19].

## 5. Frailty

Diabetes is associated with an increased risk of frailty. The Beijing longitudinal study of ageing II (BLSA-II) found the prevalence of frailty to be highest in people with diabetes (19.32%) compared to people without diabetes (11.92%). The prevalence increased with increasing age {highest in the oldest (≥85 years) 42.31%}, with increasing morbidities (highest in people with ≥3 morbidities, 49.34%) and with an increased number of medications (highest in people taking ≥4 medications, 36.19%) [20]. In a systematic review of 39 (mostly cross-sectional) studies, the prevalence of frailty in older people with diabetes was 30.0% (95% CI 23.6% to 36.7%) and pre-frailty 45.1% (38.5% to 51.8%). The identified risk factors were increasing age (OR 1.08, 95% CI 1.04 to 1.13, *p* < 0.05), sedentary life (3.11, 1.36 to 7.12, *p* < 0.001), and poor glycaemic control (2.14, 1.30 to 3.50, *p* < 0.001) [21]. Although frailty increases proportionally with increasing age and number of morbidities in older people with diabetes, the relation of frailty and blood glucose seems to be more complex. A prospective study using continuous glucose monitoring found that not only do episodes of hyperglycaemia increase the risk of frailty, but so does the time spent above range, especially above 13.9 mmol/L [22]. Equally, in another prospective study, hypoglycaemic episodes were independently associated with an increased risk of incident frailty (HR 1.44, 95% CI 1.01 to 2.05) [23].

## 6. Institutionalisation

A simulation model, based on data from the National Health and Nutrition Examination Survey (NHANES) I Epidemiologic Follow up Study, found that diabetes was responsible for 52.1% of institutionalisation [24]. Predictors of institutionalisation of older people with diabetes included mild, moderate and severe physical dysfunction {ORs and 95% CIs are 3.27 (2.60 to 4.19), 8.48 (6.02 to 13.09), and 12.53 (8.03 to 19.98)}, cognitive dysfunction (2.00, 1.60 to 2.68), and stroke (2.08, 1.61 to 2.80). Functional impairment was the main driver of increasing costs [25]. Because of the increased prevalence of diabetes in old age and increasing life expectancy, the prevalence of care home residents with diabetes is increasing. Previous studies showed that around a quarter of care home residents in the US had diabetes [26]. This has increased recently to almost one third of residents in US care homes [27]. In Canada, the prevalence of diabetes peaked at 42.1% in some areas [28]. In Europe, a 21.8% prevalence was reported in 59 nursing homes from eight countries [29]. However, the real global prevalence in care homes could be higher, as these reported figures were for people known to have a diabetes diagnosis on admission, but regular follow-up screening for diabetes development in these settings is not consistent [30].

## 7. Diagnosis

Compared with younger people, the diagnosis of diabetes in older people may not be straightforward due to the non-specific symptoms in old age. Older people with diabetes may not have any specific symptom of diabetes or present with increasing fatigue, malaise, weakness, lethargy, confusion and weight loss, which may be misdiagnosed as ageing-related. Other diagnostic difficulties include the confusion of urinary symptoms of diabetes with prostate problems in men or recurrent urinary tract infections (UTI) in women. The typical osmotic symptoms of diabetes may not be prominent, such as polydipsia, due to reduced thirst sensation, and polyuria, due to increased glucose renal threshold with increasing age [31]. Patients may also first present with common geriatric conditions such as falls and incontinence or with a diabetes-related complication such as hyperosmolar hyperglycaemic state (HHS). In asymptomatic patients, diabetes may be diagnosed during a routine blood test. The diagnosis of diabetes based on blood glucose levels criteria are the same as for younger people. However, in old age, fasting blood glucose is less sensitive and may be normal in early stages of diabetes. The 2 h glucose tolerance test may be diagnostic in these cases [32]. HbA1c is a convenient diagnostic test with high specificity (98.7%) but it can miss cases due to its low sensitivity (46.8%) [33]. It is important to take into consideration that HbA1c increases with age independent of glucose. This suggests that there may be other factors contributing to HbA1c levels and may lead to the over-diagnosis of diabetes [34]. Similarly, HbA1c can be affected by anaemia such as iron deficiency, which increases its levels [35].

## 8. Management

### 8.1. Lifestyle Intervention

The adoption of a healthy lifestyle is important for the success of pharmacologic intervention and glycaemic control. Randomised controlled trials showed that intensive lifestyle intervention (ILI) of exercise and weight reduction was successful in preventing diabetes in subjects without diabetes and in improving the metabolic profile, including cognitive function, in subjects with diabetes [36,37,38]. There was also benefit in the outcomes relevant to older people such as improved physical function, quality of life, and reduced morbidities in follow-up studies [39,40,41,42]. A high-quality protein diet, rich in essential amino acids, is required in older people with diabetes to overcome the anabolic resistance associated with ageing [43]. The European Union Geriatric Medicine Society (EUGMS), in cooperation with other scientific organisations, recommends at least 1.0–1.2 g/kg body weight daily protein, but a higher intake of up to 2.0 g/kg may be required when severe malnutrition exists [44]. A randomised controlled trial showed that progressive resistance training (PRT) exercise improved muscle quality and muscle mass compared to usual care [45]. Other clinical and observational studies showed that a diet rich in proteins combined with PRT exercise improved muscle strength and performance [46,47]. A diet rich in fruits and vegetables may help reduce the risk of frailty, as reported in a prospective cohort study [48]. A meta-analysis of 34 randomised controlled trials showed that educational programmes that provide older people with advice on a healthy diet, reduced alcohol consumption, and smoking cessation might improve glycaemic control and metabolic profile [49].

### 8.2. Pharmacologic Intervention

Because of the heterogeneous nature of older people with diabetes, the choice of glucose-lowering therapy should be personalised, especially in frail individuals. With the development of frailty, the metabolic profile of patients with diabetes spans across a spectrum from anorexic malnourished (AM) individuals at one end to sarcopenic obese (SO) individuals at the other end [50]. The AM phenotype is characterised by significant weight loss and, as a result, the regression of cardiovascular risk factors, reduced insulin resistance, reduction in HbA1c, and in severe cases, complete resolution of hyperglycaemia, a condition termed burnt-out diabetes [51]. On the other hand, in the SO phenotype, there is progression of the metabolic syndrome, cardiovascular risk factors, increased insulin resistance, and persistent hyperglycaemia. So, glucose-lowering agents can be used based on their effect on body weight.

#### 8.2.1. Weight Neutral Agents

Metformin and DPP-4 inhibitors: Both metformin and dipeptidyl peptidase-4 (DPP-4) inhibitors are well-tolerated agents with no significant effect on body weight and a low risk of hypoglycaemia. Clinical trials showed that metformin reduces the risk of cardiovascular (CV) mortality and the development of frailty in comparison to sulfonylureas (SUs) [52,53]. In a cross-sectional study, metformin therapy was independently associated with a lower risk of physical frailty in 736 community-dwelling veterans (≥65 years old) with type 2 diabetes [54]. In a cohort study, metformin showed a pleotropic effect by slowing down the development of age-related comorbidities such as cardiovascular diseases, cancer, depression, dementia, and frailty [55]. However, it has been reported that metformin may induce vitamin B_12_ deficiency, which may worsen cognitive function, and the addition of vitamin B_12_ supplements to metformin therapy may have a cognitive protective effect [56]. In a prospective study, metformin improved cognitive function and depressive symptoms in patients with comorbid type 2 diabetes and depression compared with placebo [57]. The DPP-4 inhibitors lack the CV benefits of metformin, but they are a good choice in older people because of their tolerability [58]. Although DPP-4 inhibitors have less prognostic benefits compared to metformin, they may protect cognitive function [59]. In a real-world population-based study, DPP-4 inhibitor therapy reduced the risk of all-cause dementia in comparison to SU [60]. In a case–control study, treatment with DPP-4 inhibitors was associated with a lower risk of depression in patients treated for diabetes compared to patients with diabetes but not on medication [61].

#### 8.2.2. Weight-Loss Agents

Acarabose, SGLT-2 inhibitors, and GLP-1RA: Acarabose delays carbohydrate absorption in the small intestine, reducing postprandial blood glucose peaks. It has some CV benefits, like a low risk of hypoglycaemia, and it promotes weight loss, but it is less tolerated due to gastrointestinal side effects [62]. Randomised controlled trials showed that sodium glucose co-transporter-2 (SGLT-2) inhibitors and glucagon-like peptide-1 receptor agonists (GLP-1RA) reduce body weight and have a low risk of hypoglycaemia. They have a significant cardio-renal protective effect, which extends to include older people with diabetes [63]. A prospective study showed the beneficial effects of the SGLT-2 inhibitor empagliflozin on physical and cognitive impairment in frail older people with diabetes and heart failure [64]. GLP-1RA may have potential to reduce the risk of cognitive impairment [65]. The large prospective Researching Cardiovascular Events With a Weekly Incretin in Diabetes (REWIND) trial reported a 14% reduction in the risk of cognitive impairment by the GLP-1 RA dulaglutide [66].

#### 8.2.3. Weight-Gain Agents

Insulin secretagogues, glitazones and insulin: Insulin secretagogues such as SU and glinides, to a lesser degree, are associated with weight gain and increased risk of hypoglycaemia, and their CV effects are conflicting [67]. There is limited evidence to suggest that SU may increase the risk of depression, dementia, and muscle atrophy [61,68,69]. They are not a preferred choice in older people. Glitazones have a low risk of hypoglycaemia, but they increase body weight. Glitazones may have favourable CV benefits, but they mildly increase the risk of volume overload in people with heart failure [70]. They may also reduce muscle mass loss and preserve walking speed [71]. Prospective studies showed that glitazones attenuated the decline in the loss of walking speed, and reduced the risk of CV risk events and dementia, but increased the risk of fractures in older people with diabetes [72,73]. Insulin is the most effective hypoglycaemic agent, but it increases body weight and the risk of hypoglycaemia [74]. Insulin has also been shown to increase the risk of dementia, although authors suggested that repeated episodes of insulin-induced unreported hypoglycaemia might have mediated an increased risk of dementia [75]. Insulin has no CV benefits, and although it has anabolic properties, its effects on muscle mass and frailty need future exploration [76]. Studies investigating the effects of glucose-lowering agents on the multidimensional aspects of frailty are only few, as frailty was not commonly addressed in outcome trials. The effects of glucose-lowering agents on outcomes relevant to older people are summarised in Table 1.

### 8.3. Therapeutic Strategy

**Figure 1:**
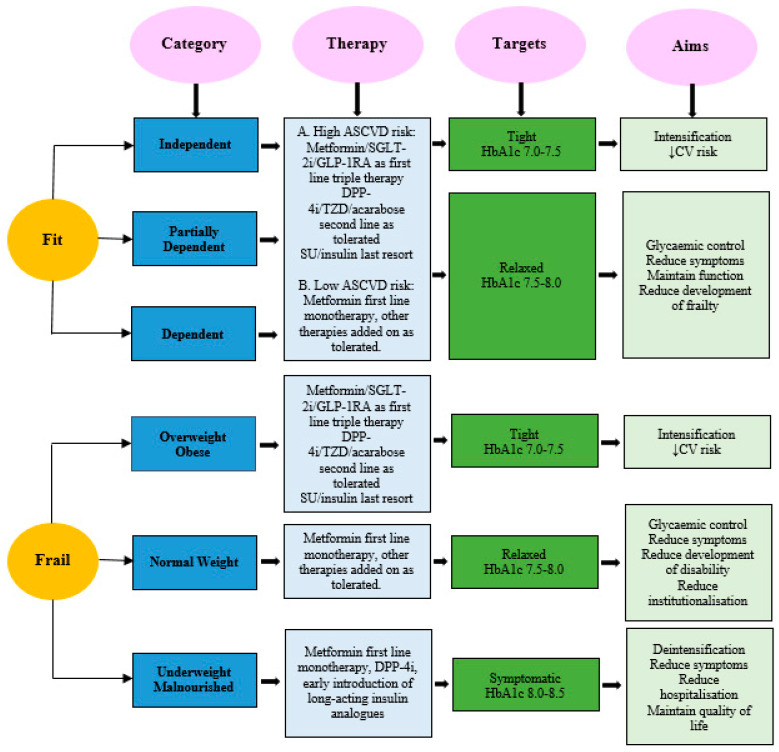
A stepwise approach of glucose-lowering agents, hypoglycaemic targets, and aims of therapy based on patients’ categories, classified according to functional fitness and frailty phenotypes. Independent = No help, Partially dependent = Some help, and Dependent = All help, ASCVD = Atherosclerotic cardiovascular disease, SGLT-2i = Sodium glucose cotransporter-2 inhibitors, GLP-1RA = Glucagon like peptide-1 receptor agonists, CV = cardiovascular, DPP-4i = Dipeptidyl peptidase-4 inhibitors, TZD = Thiazolidinediones, SU = sulfonylureas. Overweight = BMI > 25 kg/m^2^, Obese = BMI > 30 kg/m^2^, Normal weight = BMI = 18.5–25 kg/m^2^, and Underweight = BMI < 18.5 kg/m^2^. The stepwise approach should be dynamic following the changes in patients’ functional condition and body composition in both directions.

Metformin remains the first-line glucose-lowering agent for most patients due to its safety and prognostic benefits. The following steps of therapy choice depend on the underlying atherosclerotic cardiovascular disease (ASCVD) risk and frailty phenotype. Glycaemic targets will depend on overall prognosis and the likely benefits. This is linked to the underlying morbidities, function, and life expectancy. (Figure 1).

Fit individuals: For fit individuals, a triple first-line therapy of metformin, SGLT-2 inhibitors, and GLP-1RA is recommended by major guidelines for patients with high ASCVD risk such as the presence of multiple CV risk factors, heart failure, or chronic kidney disease (CKD) [77,78]. Although the guidelines are not explicit for triple therapy in older individuals, such a recommendation can be adopted in fit older people on a cautious case-by-case approach. The initiation of triple therapy in gradual steps with metformin as a first line, followed by SGLT-2 inhibitors and then GLP-1RA, is appropriate. Tolerability should be closely monitored to avoid side-effects, and stepping down may then be required. Other therapies with low hypoglycaemic risk, such as DPP-4 inhibitors, glitazones, or acarabose, are second-line therapy as tolerated. SU and insulin are a last resort due to the risk of hypoglycaemia. For independent subjects, tight glycaemic targets of HbA1c 7.0–7.5% are reasonable, and relaxed targets of HbA1c 7.5–8.0 are more suitable for partially dependent and dependent subjects. The main aim is the intensification of therapy to reduce CV risk in independent subjects. In partially dependent and dependent subjects, glycaemic control to reduce complications, maintain function, and reduce the development of frailty are the main aims.

Frail individuals: For SO individuals, the synergistic effects of sarcopenia and obesity increases insulin resistance and CV risk more than each condition alone [79]. Therefore, the triple first-line therapy of metformin, SGLT-2 inhibitors, and GLP-1RA is indicated in this phenotype to improve CV outcomes. This phenotype has been shown to benefit most from SGLT-2 inhibitors and GLP-1RA therapy due to their higher baseline CV risk compared to non-frail individuals [80]. Therefore, triple therapy can be tried in patients with BMI ≥ 18.5 kg/m^2^, only if tolerated, and the benefit is likely to increase proportionally with increasing body weight. On the other hand, due to significant weight loss in the AM phenotype and reduced insulin resistance, the early use of long-acting insulin analogues is more appropriate, due the benefits of the anabolic properties of insulin. We suggest a body mass index (BMI) <18.5 kg/m^2^ as the definition of underweight. Although the accuracy of BMI poses a challenge in old age, a BMI of <18.5 is likely to be a true reflection of being underweight, as it is already overestimated due to the loss of height because of osteoporotic vertebral compressions, intervertebral disc space narrowing, and kyphosis associated with ageing. In this phenotype, deintensification of therapy and a focus on prevention of hospitalisation and maintenance of quality of life are the aims of therapy.

### 8.4. Other Therapies

Older people with diabetes will benefit most from cholesterol-lowering agents compared with younger people, due to their high baseline ASCVD risk [81]. Current evidence suggests that the benefit includes older people up to the age of 85 years, but it attenuates in the very older age groups [82,83,84,85]. Lipid-lowering therapies such as statins, ezetimibe, fibrates, bempedoic acid, and proprotein convertase subtilisin/kexin type 9 (PCSK9) inhibitors can be used individually or in combination as tolerated. The targets are to reduce LDL to <1.4 mmol/L in secondary prevention and <1.8 mmol/L in primary prevention [77]. The benefits of blood pressure (BP) lowering are significantly higher in older people with diabetes compared to those without diabetes, due to their higher baseline CV risk [86]. A BP target of <130/80 mmHg is recommended if it can be safely attained [77]. Relaxed targets are more suitable in patients with multiple morbidities and less tolerance to polypharmacy. Most older patients will need more than a single antihypertensive agent, and the recent Quadruple Ultra-Low-Dose Treatment for Hypertension USA (QUARTET USA) trial has shown that the use of ultra-low-dose quadruple-combination therapy reduced BP more effectively with fewer side effects, compared with a standard-dose monotherapy [87]. Antiplatelets are recommended for secondary prevention. They can be used for primary prevention in patients with high ASCVD and low bleeding risks [77].

## 9. Special Considerations in Old Age

### 9.1. Hypoglycaemia

In older people with diabetes, hypoglycaemia is particularly important due to difficulties in recognition and the associated serious consequences. In old age, neurologic, rather than autonomic, symptoms of hypoglycaemia are more dominant. Non-specific symptoms such as dizziness and feeling generally unwell may delay diagnosis [88]. Hypoglycaemia presenting with confusion or agitation may be attributed to ageing or cognitive impairment. In a primary care study, nausea, falls and unsteadiness were identified as common non-specific symptoms of hypoglycaemia [89]. As a result, hypoglycaemia may be under-reported in older age groups [90]. Hypoglycaemia is associated with an increased risk of physical and cognitive dysfunctions as well as serious cardiac events [91]. The risk of hypoglycaemia increases with increasing age, morbidities, and polypharmacy (Box 1). Therefore, hypoglycaemia should be suspected in older people with diabetes who are feeling unwell or confused with no obvious other causes. Patients and their carers should be made aware of the atypical presentation of hypoglycaemia, and ensure frequent and small meals, especially for patients with erratic eating patterns or having underlying cognitive dysfunction. Short-acting insulin, delivered after meals, is safer than before meals to avoid the risk of hypoglycaemia if food is not consumed. As a rule, the choice of hypoglycaemic agents with low hypoglycaemic potential should be considered first, and agents such as SU are better avoided especially in frail AM patients. Polypharmacy should be reviewed to reduce the number of medications as much as possible, to lessen drug interactions, and complex hypoglycaemic regimens should be simplified. In addition, regular monitoring of renal and liver functions is required to adjust the appropriate doses of hypoglycaemic agents. Furthermore, glycaemic targets and goals of therapy should be periodically reviewed, especially when frailty of the AM phenotype develops, which increases the risk of hypoglycaemia. The social factors associated with hypoglycaemia risk such as isolation and a lack of support should be looked at regularly.

Box 1 **Risk factors for hypoglycaemia in older people with diabetes.**

Increasesing

- Age
- Diabetes duration
- Morbidities
- Hospitalisations
- Polypharmacy
- Drug errors
- Hypoglycaemia potentiating agents
- Organs dysfunction
- Erratic eating
- Malnutrition

Decreasesing

- Body weight
- Cognitive function
- Physical function
- HbA1c
- Hypoglycaemia awareness
- Counter regulatory hormones
- Typical symptoms of hypoglycaemia
- Recognition by carers or health care professionals
- Food intake
- Social support

### 9.2. Dementia

Older people with poor glycaemic control or a longer duration of diabetes have been reported to have a higher risk of cognitive decline compared to those with controlled diabetes or a shorter duration of diabetes [92]. Repeated episodes of hypoglycaemia, which may cause cerebral neuronal damage, is associated with an increased risk of dementia in a dose–response manner. The risk of dementia increases with the increased severity and frequency of the hypoglycaemic episodes [93]. The development of dementia will have a direct clinical impact on patients’ self-care abilities and increases their risk of hypoglycaemia due to reduced or erratic eating [94,95]. Therefore, periodic screening for cognitive function in older people with diabetes should be integrated in their annual review charts, as cognitive impairment may develop gradually and not been noticed by patients’ carers [96]. Oral medications should be reviewed and the simplification of insulin regimens is required [97]. People with advanced dementia are likely to be frail, with gradual weight loss and reduced life expectancy due to reduced eating. Therefore, long-term glycaemic control may be less relevant in this cohort. A short-term blood glucose level in a comfort zone between 4 and 15 mmol/L may reduce malaise and maintain wellbeing, as values outside this range are likely to be associated with cognitive changes [98].

### 9.3. Continuous Glucose Monitoring

Continuous glucose monitoring (CGM) tracks interstitial glucose levels continuously, instead of using intermittent finger prick needles. Therefore, it provides continuous, real-time blood glucose levels non-invasively. There is increasing evidence to suggest that CGM is acceptable and manageable by older people with diabetes [99]. There is also emerging evidence that it can be successful in patients with cognitive impairment if support and education from carers are available [100]. Furthermore, it has been shown to be useful in care home settings to identify out-of-range readings and reduce the number of hypoglycaemic episodes [101,102]. CGM appears to reduce the risk of hospitalisations due to diabetes complications, diabetic ketoacidosis (DKA), and diabetes-associated comas in older people with both type 1 and type 2 diabetes [103]. It also has the advantage of detecting non-symptomatic hypoglycaemia, which is common in older people regardless of HbA1c levels [104]. Patients with type 1 diabetes or insulin-treated type 2 diabetes will benefit from CGM, but it is not required in patients on oral hypoglycaemic therapy [105]. CGM is also useful in complex older people with morbidities. In a study involving a cohort of both type 1 and type 2 older people with diabetes—mean (SD) age 73.3 (6.2) years, Charlson comorbidity index score 5.6 (1.5), duration of diabetes 31.3 (15.4) years, and clinical frailty score 4.6 (2.3)—CGM significantly improved HbA1c without an increase in the time spent in hypoglycaemia [106]. The overall evidence so far supports the use of CGM in a broad range of older people with diabetes, from fit to frail or from healthy to those with complex health conditions, due to its benefits for all aspects of diabetes care and improving patients’ quality of life. However, there is no clear guidance on CGM metrics specific for older people. The metrics for older people should focus on avoiding hypoglycaemia and targets tailored according to physical function. Our suggested metrics are summarised in Table 2. There is no clinical trial evidence on CGM time in range, and clinical practice follows expert opinion. We have kept the required time in range in line with the recommendations from the international consensus on time in range, but adapted the targets according to patients’ function [107].

### 9.4. Mobile Health

With advances in technology and increasing access, there is an increasing scope for the use of mobile health (mHealth) in diabetes management and self-care. It may have the potential to ease the burden on patients’ carers and improve other aspects of diabetes care, such as compliance and glycaemic control [108]. The increased use of mobile phones by older people will help the use of different software applications relevant to glucose monitoring and diabetes care [109]. The current evidence from systematic reviews suggests that the use of mHealth app-based interventions may reduce HbA1c with a good rate of satisfaction [110,111]. A review of mHealth use in older people with diabetes demonstrated a beneficial effect on glycaemic control, physical activity, blood pressure, and lipid profile, but questioned the feasibility of its widespread implementation in older people in clinical practice [112]. However, a more recent meta-analysis of randomised controlled trials suggested that mHealth interventions might enhance diabetes-related outcomes in older people. The size effect was similar to younger people, suggesting a promising potential for mHealth use in chronic conditions such as diabetes in old age [113]. Another recent meta-analysis reported improved cardiometabolic parameters and medication adherence with mHealth intervention, compared to usual care, in older people with diabetes [114]. There is still a need for an expansion of the use of mHealth in older people, as their acceptance has been demonstrated to be influenced by the ease of use of the technology, whether there are any additional benefits, the availability of social support, app design, and eHealth literacy [115,116]. The use of technology in telemedicine is not inferior to face-to-face consultation but does not seem to provide extra benefits [117]. A large US survey found that around 50% of US adults with diabetes used telehealth, and patients reported telehealth care quality to be similar to face-to-face visits, but efforts are still required to address barriers to use [118].

### 9.5. Artificial Intelligence

The diversity and heterogeneity of older people with diabetes propose a challenge for delivering individualised care plans in conventional and traditional clinical practice [119]. A recent systematic analysis explored the use of artificial intelligence (AI) in health care, integrating machine-learning algorithms, intelligent monitoring systems, and natural language processing into the management of older people with diabetes. The evidence suggests a possible 25% decrease in hospitalisation and a 30% increase in medication adherence rates compared to conventional approaches. The analysis also suggests that AI-based clinical decision support systems (CDSSs) may provide a diagnostic accuracy of 93.07%, in addition to a superior performance in the prediction of risks and optimisation of treatment. However, improvements are still required in data standardisation, algorithm transparency, patients’ privacy protection, and multidisciplinary collaboration among technology developers, health care professionals, and regulatory authorities for the further development of AI in clinical practice [120]. The current evidence is compromised by the heterogeneity in the designs of the studies included, the AI technologies used, and population characteristics, which limits direct comparisons and the generalisability of the findings. It is suggested that CDSS improves clinicians’ confidence and patients’ trust by up to 73% and reduce medication errors by 50% [121,122]. There is however, a need to reduce the opacity of the decision-making process and improve the transparency and explanatory power of the AI models for the ease of older people’s interpretation. AI predictive abilities may aid the early identification of high-risk patients, allowing early and proactive interventions, which may improve clinical outcomes [123]. It has been highlighted that older people with diabetes will need unique AI systems and specific algorithms aligned with their needs [124]. Automated adherence reminders and adherence monitoring, advanced sensors, and wearable devices are examples of machine learning algorithms that can detect subtle physiologic changes with 81.05% sensitivity, 88.3% specificity, and 84.7% overall accuracy [125,126]. For example, in older people with diabetes, AI-based predictive models may be able to forecast adverse events such as hypoglycaemia, which helps early communication with families or carers to facilitate timely interventions, as well as the possible early detection of other diabetes-related complications [127,128]. The development of advanced tools, relevant to older people, may also aid in the comprehensive assessment of cognitive, physical, and psychological functions [120]. The improvement in prediction and patient care may reduce overall health care cost [129]. Quality of life improvement, physical independence in activities of daily living, and high satisfaction of care have been reported [130]. In summary, AI is an emerging technology that still needs further developments, refinement, and adaptation for clinical practice use in older people with diabetes.

### 9.6. Care Delivery

With increasing age and complexity of diabetes complications and morbidities, older people have complex needs and require organised care to cope with the challenges of safe diabetes management. On annual review, physical and cognitive functions should be assessed along with depression, anxiety, and self-care ability. Family support is an initial step in some patients, such as those with cognitive impairment, as this may help to keep diabetes under control [131,132]. Educational programmes tailored for carers and patients’ cognitive abilities may help control diabetes and maintain self-care [133]. When the need for care arises, organisation and good communications between different professionals is vital for good delivery of care [133]. Shared care protocols between primary and secondary care may help smooth communications and avoid the breakdown of care. In addition, a flexible service that is sensitive to patients’ needs and their changing medical condition may help collaborations between different parties [134]. Continuity of care with the same family physician is another aspect that improves patients’ experience and reduces acute hospitalisation [135]. Patients discharged from hospital should have a clear after-discharge plan communicated well to primary care to reduce chances of readmission [136]. In the community, follow-up telephone support or a nurse case manager specialising in diabetes improve the outcome and quality of life of patients [137,138]. Caring for carers is another important aspect of care delivery. Carers’ burden should be periodically checked regarding their health and any deterioration in their ability to care for patients [139]. Carers’ health is of paramount importance, as undetected deterioration in their health may lead to poor diabetes care for patients, worsening of their condition, and institutionalisation [140]. It has been shown that carers looking after older people with diabetes are commonly overburdened, and reducing that burden may improve outcomes [141,142]. Therefore, flexible and sensitive plans for carers’ needs should be in place from the outset, which respond to their requirements in a timely way to help reduce the deterioration of their health and burden of care [143]. The main points required in annual reviews for older people with diabetes are summarised in Box 2.

Box 2 **Main annual review points in older people with diabetes.**

Patient’ details: Age, gender, body weight, height, BMI, waist circumference.

Lifestyle: Healthy diet, ideal body weight, smoking cessation, alcohol reduction.

Activities of daily living: Washing, dressing, mobility, feeding, toilet use, continence, shopping, finance, driving.

Medical history: Morbidities, medications, recent blood results.

Vascular disease: Macrovascular, microvascular.

Foot inspection: Circulation, sensation, infections.

Screen: Frailty, cognition, depression, physical function.

Self-care: Ability of self-care, carer support, social factors.

General: Nutrition, hydration, oral hygiene, dental care, sexual function.

Education: Periodic programmes for patients and carers, carers’ burden.

Prevention: Vaccination programmes, sick day rules.

Future planning: Goals of therapy, reduction in polypharmacy, hospitalisation avoidance, deintensification, terminal care.

## 10. Reduction in Therapy Burden

Reduction in therapy burden should be considered at some point when a patient’s condition becomes more complex, with reduced coping ability due to complex regimens or an increased incidence of therapy-related complications.

### 10.1. Deintensification

Overtreatment should be avoided in older people with diabetes, especially those with increasing age and multimorbidity burden, as this may lead hypoglycaemia and hospitalisations [144]. The development of frailty, in addition to multimorbidities, is another factor to review medications and therapeutic goals. It is crucial to consider frailty metabolic phenotypes when deintensifying hypoglycaemic therapy. For example, deintensification, especially of high hypoglycaemia risk medications, should be considered in the AM frailty phenotype who have significant weight loss and high risk of hypoglycaemia. The weight loss may lead to normalisation of hyperglycaemia, reduction in HbA1c and a state of burnt-out diabetes [145]. Patients with significant anorexia should be considered for deintensification or even complete withdrawal of hypoglycaemic therapy [146]. On the other hand, medications such as SGLT-2 inhibitors and GLP-1RA should be continued in the SO frailty phenotype due to their cardioprotective effect. In the SO frail phenotype or non-frail patients with high ASCVD risk, statin therapy should be continued, as discontinuation in this group may lead to adverse CV outcomes [147]. Statins however, do not appear to have benefits in the very old (>85 years) without ASCVD risk or those living in care homes specially the AM frail individuals with limited life expectancy [148,149]. Although anti-hypertensive medications should be continued in patients with high ASCVD risk, they can be reduced to maintain a relaxed target in the AM frail individuals who tend to have low BP due to weight loss. A reduction in medications to keep blood pressure around 150/90 mmHg in this group of patients is reasonable [150]. Aspirin should be continued in those with high ASCVD risk but in primary prevention, the benefits outweigh the risk of bleeding and should be considered on a case-by-case basis [151].

### 10.2. Palliation and Terminal Care

**Figure 2:**
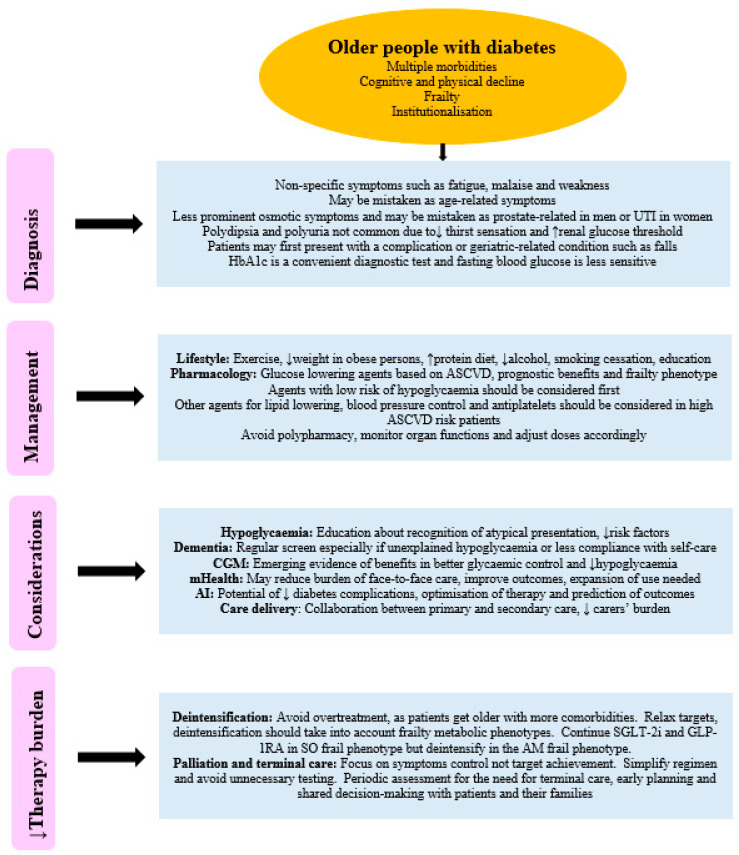
A holistic approach to the management of diabetes in old age. UTI = urinary tract infection, ASCVD = Atherosclerotic cardiovascular disease, CGM = Continuous glucose monitoring, mHealth = Mobile health, AI = Artificial intelligence. SGLT-2i = Sodium glucose cotransporter-2 inhibitors, GLP-1RA = Glucagon like peptide-1 receptor agonists, SO = Sarcopenic obese, and AM = Anorexic malnourished.

At some stage with further progression of age and disability, the aim of therapy should focus on palliation, symptom relief, and preparation for terminal care, rather than target achievement. Health care professionals should be aware of patients approaching their palliative phase of life, as this may not be very clear [152]. Older people with diabetes near this phase commonly have complex conditions that lead to over-utilisation of health care resources, frequent hospital admissions, and reduced quality of life [153]. Palliation strategies include simplification of regimens, reduction in frequent blood glucose testing, avoiding invasive interventions, and focusing on the maintenance of blood glucose in a comfort zone with little fluctuations to avoid emergency hospitalisations. Planned, rather than acute, palliation is more appropriate with patients and their carers’ consultations to avoid suffering [154]. A multidisciplinary approach to palliation, which includes comprehensive geriatric assessment, periodic monitoring of patient condition, and timely identification of progression to terminal care, is appropriate. Terminal care should be recognised when life expectancy is less than one year due to either diabetes or other associated morbidities to avoid delays in providing care [155]. The surprise question (SQ) “Would you be surprised if this patient died within the next X months?” may help clinicians’ identification of patients approaching the terminal phase of life [156]. The SQ reached 75.4% accuracy in one systematic review [157]. It is a simple way to predict prognosis, as so far, there are no accurate tools to help predict approaching death in palliated patients [158]. Medications should focus on comfort and pain relief. In the last days of life, especially when patients’ eating patterns are erratic, oral hypoglycaemic therapy can be completely withdrawn in patients with type 2 diabetes and replaced by basal once daily, as well as short-acting insulin given as required, to keep blood glucose levels in the comfort non-symptomatic zone. In patients with type 1 diabetes, insulin can be continued at a reduced dose. In the last hours of life or when patients are unconscious, insulin can be completely stopped in both types of diabetes. A holistic approach to diabetes care in old age is illustrated in Figure 2.

## 11. Conclusions

Diabetes prevalence is increasing, particularly in the older age group. Older people with diabetes are clinically complex with multiple physical and cognitive morbidities that lead to frailty, disability, and institutionalisation. Therefore, the management of this complex and heterogeneous cohort should take into considerations this wide variability. Clinicians require skills and expertise in geriatric assessment and a heightened awareness of the syndromes of age-related disease. Strict targets are appropriate in independent individuals but should be gradually relaxed when a decline in function and complexity of overall health condition occurs. The development of frailty is an important factor, as the sarcopenic obese phenotype, although frail, will still benefit from agents that reduce cardiovascular events due to high baseline cardiovascular risk. With the progression of age and development of disability and reduced life expectancy, deintensification of therapy should be considered, especially in the anorexic malnourished frailty phenotype due to significant weight loss and high risk of hypoglycaemia. In this palliative phase of life, family communication and shared decision-making should start early about planning for terminal care.

## 12. Future Perspectives

The management of diabetes in old age is challenging due to advanced age and disease, and the competing morbidities leading to variations in medication tolerance. In addition, the development of frailty changes the metabolic profile of individuals and their diabetes trajectory [159]. Furthermore, outcomes relevant to older people, such as frailty, with its wider dimensional aspects of physical and cognitive functions, and depression, are not commonly addressed in diabetes outcome studies. Therefore, future clinical trials that address older age-related outcomes are required. The frailty metabolic spectrum needs further exploration. Individual frailty phenotypes should be characterised from the outset of clinical trials to specifically investigate outcomes relevant to the underlying metabolic profile. Future glucose-lowering agents are moving away from just glucose-lowering into wider cardiovascular and extra glycaemic properties, which will have a more positive effect than the traditional hypoglycaemic agents. The cardio-protective effects of SGLT-2 inhibitors and GLP-1RA may have a long-term impact on improving the overall function and reducing the development of frailty, but this requires further investigations.

For example, there is emerging evidence that these agents may reduce the risk of the development of Alzheimer’s and Parkinson’s diseases [160,161]. However, their direct effect on muscle mass is not clear, and whether they cause sarcopenia and increase the risk of frailty needs future exploration. The introduction of CGM has improved outcomes and quality of life, but its use in older people is suboptimal at present. There is still no clear guidance on targets specific for older people. We have suggested some guidance based on the functional category and health status of older people, but this needs to be tested and further refined in real-world studies. Challenges such as dexterity issues, sensory impairments, technology literacy, and compliance need to be addressed in future devices, which should be tailored for older patients and their carers. With the increasing number of older people with diabetes and increasing life expectancy, the introduction of mHealth may help overwhelmed health care systems. Future research and devices should address barriers such as digital abilities, physical function, and cognitive decline, which may affect the expansion of use. In addition, positive promotion to gain users’ trust and increase motivation, and the removal of technical barriers with appropriate training, are still required. Similarly, the introduction of AI is an innovation that has the potential to improve care for older people and improve the health system’s operating efficiency. However, its use in older people may be complex, and improvements in usability, accessibility, and data privacy are needed. Comprehensive care for older people with diabetes should not only focus on the intensification of therapy, but also should take into account when it is appropriate to consider deintensification and palliation. The criteria of patients suitable for deintensification and a pathway for deintensification are not clearly defined in clinical guidelines. Future studies on patients’ characteristics and deintensification strategies are important. For example, we have suggested that agents with good prognosis, such as SGLT-2 inhibitors and GLP-1RA, should not be deintensified in the SO frailty phenotype but only in the AM phenotype. Therefore, future studies examining the prognostic benefits of glucose-lowering therapy in frail individuals based on their metabolic phenotype is required. The anabolic properties of a long-acting insulin analogue given daily or weekly in the AM frailty phenotype needs exploration, as well as its impact on quality of life. Deintensification of therapy should be associated with early plans for palliation and terminal care. Clinicians’ early prediction of the terminal phase of life needs improvement, and the development of predictive tools is required to avoid unnecessary end-of-life hospital admissions. Research is needed to address the barriers for making end-of-life decisions in the community and to facilitate smooth diabetes care in this terminal phase, including family support to reduce their stress and maintain the quality of the last days of life (Box 3).

Box 3 **Future research directions.**

**What is already known**

- Ageing of the population is increasing due to increased life expectancy.
- Diabetes prevalence is proportionally increasing with increasing age.
- Older people with diabetes are heterogeneous with a complex condition.
- Current management of diabetes in old age is challenging.
- No direct evidence for diabetes-related outcomes in this age group.

**Future directions**

- The development of frailty changes body composition and insulin resistance.
- The metabolic impact of frailty on diabetes trajectory needs exploration.
- Frailty metabolic profile should be determined from the outset in future studies.
- Old age-related outcomes such as physical frailty, cognition, and depression should be included in future studies.
- The effects of SGLT-2 inhibitors and GLP-1RA on these outcomes is required, especially the effect on muscle mass.
- Novel agents with extra glycaemic effects on these outcomes are still required.
- The introduction of CGM is promising in reducing the risk of hypoglycaemia, and research to overcome barriers of use is needed.
- mHealth may help manage the increasing number of older people with diabetes and reduce the need for face-to-face appointments.
- The emergence of AI may help improve outcomes, but it needs improvement to increase trust and patients’ security.
- Clinical pathways and strategies are still needed to recognise patients who need deintensification, withdrawal of therapy, and palliation.

SGLT-2i = Sodium glucose cotransporter-2 inhibitors, GLP-1RA = Glucagon like peptide-1 receptor agonists, CGM = Continuous glucose monitoring, mHealth = Mobile health, and AI = Artificial in-telligence.

### Key Points

- The prevalence of older people living with diabetes is increasing.
- Older people with diabetes are complex and their management is challenging.
- The development of frailty affects the metabolic profile and the choice of glucose-lowering agents.
- Intensification of therapy is required in the sarcopenic obese frail, while in the anorexic malnourished frail, deintensification of therapy is appropriate.
- Future research for the expansion of technology use in the management of diabetes in old age is still required.

**What this review adds**

This review represents a comprehensive and up-to-date account of the current evidence of the management of older people with diabetes.It highlights the heterogeneity of this population and draws attention that frailty changes the metabolic profile of individuals and shifts diabetes trajectory.It aims to move beyond age-based management and propose a phenotype-driven framework integrating functional status, frailty phenotypes, vascular risk, and dependency level into a therapeutic decision-making process.It investigates the current evidence in diabetes care-related technology and suggests personalised metrics based on older person function and morbidities.The review suggests a logarithmic personalised approach of choice of hypoglycaemic therapy based on biologic foundation rather than chronological age.


## Figures and Tables

**Table 1:** Effects of glucose-lowering therapy on aspects relevant to older people.

Agent	Body Weight	CV Safety	Depression	Frailty	Dementia	Cautions
Metformin	Neutral.	Modest CV benefit.	Likely positive.	Likely positive.	Likely positive but effect may be limited by potential inducing vitamin B12 deficiency.	Monitor renal function, hold in acute illness.
DPP-4i	Neutral.	Neutral. Potential increase in HF hospitalisation with saxagliptin and alogliptin.	Likely positive.	Likely positive but less clear.	Likely positive.	Monitor renal function and avoid in HF with some DPP-4i.
Acarabose	Modest weight loss.	May have some benefits when added to metformin.	No data.	Likely negative but no data.	Likely positive but less data.	Withdraw if intolerant diarrhoea develops.
SGLT-2i	Significant weight loss.	Significant CV risk reduction.	Likely positive but little data.	Less clear.	Neutral but potential benefit.	Monitor renal function, hold in acute illness, avoid in AM frail.
GLP-1RA	Significant weight loss.	Significant CV risk reduction.	No clear data.	Less clear.	Neutral but potential benefit.	Monitor renal function, hold in acute illness, avoid in AM frail.
Insulin secretagogues	Modest weight gain.	Likely negative.	Likely negative but less data	Likely negative but less data.	Likely negative but less data.	Monitor for hypoglycaemia.
TZD	Modest weight gain due to fluid retention.	Modest CV benefits but increased risk of HF exacerbation.	Positive	Likely positive but less data.	Likely positive.	Avoid in HF.
Insulin	Significant weight gain.	Likely neutral.	Likely negative.	Less clear.	Less clear.	Monitor for hypoglycaemia.

CV = Cardiovascular, DPP-4i = Dipeptidyl peptidase-4 inhibitors, HF = Heart failure, SGLT-2i = Sodium glucose cotransporter-2 inhibitors, AM = Anorexic malnourished, GLP-1RA = Glucagon like peptide-1 receptor agonists, and TZD = Thiazolidinediones.

**Table 2:** CGM and targets in different subgroups of older patients based on function.

	Independent	Partially Dependent	Dependent
Target range	4–10 mmol/L	5–11 mmol/L	6–12 mmol/L
TIR	≥70%	≥70%	≥70%
TBR (<3 mmol/L)	<1% 0%	<1% 0%	<1% 0%
TAR	<20% >12 mmol/L <10%	<20% >13 mmol/L <10%	<20% >14 mmol/L <10%
CV	<36%	<36%	<36%
GMI (estimated HbA1c)	7–7.5%	7.5–8%	7.5–8.5%

Independent = No help, minimal morbidities, no polypharmacy, no organ dysfunction. Partially dependent = Some help, moderate morbidities, mild polypharmacy, mild organ dysfunction. Dependent = All help, multiple morbidities, significant polypharmacy, advanced organ dysfunction. CGM = Continuous glucose monitoring, TIR = Time in range, TBR = Time below range, TAR = Time above range, CV = Coefficient of variation (<36% = Stable glycaemia, ≥36% = Unstable glycaemia), and GMI = Glucose management index. Percentages of time in range targets are according to recommendations from the international consensus on TIR, but we adapted the targets according to patients’ function [107].

## Data Availability

No new data were created or analyzed in this study. Data sharing is not applicable to this article.
